# Improved prediction of hip fracture using multi-faceted biomechanical computed tomography

**DOI:** 10.1093/jbmr/zjaf139

**Published:** 2025-10-04

**Authors:** Tony M Keaveny, Annette L Adams, Eric S Orwoll, Sundeep Khosla, Michael R McClung, Mary L Bouxsein, Shireen Fatemi, Bryce A Besler, David C Lee, David L Kopperdahl

**Affiliations:** Departments of Mechanical Engineering and Bioengineering, University of California, Berkeley, CA 94720, United States; O.N. Diagnostics LLC, Berkeley, CA 94704, United States; Department of Research and Evaluation, Kaiser Permanente Southern California, Pasadena, CA 91101, United States; Bone and Mineral Unit, Department of Medicine, Oregon Health & Science University, Portland, OR 97239, United States; Robert and Arlene Kogod Center on Aging and Division of Endocrinology, Mayo Clinic, Rochester, MN 55905, United States; Oregon Osteoporosis Center, Portland, OR 97225, United States; Center for Advanced Orthopedic Studies, Beth Israel Deaconess Medical Center and Department of Orthopedic Surgery, Harvard Medical School, Boston, MA 02215, United States; Department of Endocrinology, Kaiser Permanente Southern California, Panorama City, CA 91402, United States; O.N. Diagnostics LLC, Berkeley, CA 94704, United States; O.N. Diagnostics LLC, Berkeley, CA 94704, United States; O.N. Diagnostics LLC, Berkeley, CA 94704, United States

**Keywords:** osteoporosis, hip fracture, fracture risk, BMD, bone strength, muscle

## Abstract

With the goal of preventing more hip fractures, a next generation of the VirtuOst Biomechanical Computed Tomography (BCT) test was developed that integrates measurements from a clinical CT scan related to fall risk, impact force, and femoral strength, the three main determinants of hip fracture. Here, we introduce the test and validate it against BMD and FRAX. Our source population from a large healthcare system comprised of 341364 patients (≥65 yr) with an abdominal-pelvic CT during care. Using data from 3035 patients (1790 with hip fracture), we developed a “BCT Risk Score” (range: 0-100) having input risk factors of age, femoral strength, ratio of trabecular/cortical BMD, muscle area, intramuscular fat, FN volume, hip width, and posterior fat thickness. In a geographically distinct set of 2124 patients (1293 with hip fracture), we then compared the BCT Risk Score against a DXA-equivalent hip BMD T-score (lowest hip value, measured from the CT scan by VirtuOst) and FRAX hip fracture risk (with BMD but without parental fracture history) for predicting a first incident hip fracture within 5 yr. For the women, the c-statistic for predicting fracture was higher for BCT (0.89, 95% CI: 0.87-0.90) than for BMD (0.81, 0.79-0.84) or FRAX (0.85, 0.83-0.87). Using binary thresholds to identify high-risk patients, sensitivity for BCT (Risk Score ≥ 75) was higher than for BMD (T-score ≤ −2.5) and FRAX (hip risk ≥ 3.0%): 81.4% vs 47.8% vs 75.9%, respectively; positive predictive values confirmed comparable high-risk status (BCT 13.6% vs BMD 15.3% vs FRAX 12.7%). Similar trends were observed for the men, 2-yr outcomes, and identifying very-high-risk patients. We conclude that, compared to both BMD and FRAX, the integrative BCT test better predicted hip fracture and its high sensitivity should improve fracture prevention.

## Introduction

More than one million fragility fractures occur annually in the United States, hip fractures being the most serious and costly.^1^^,^^2^ Today, multiple drug treatments are available that can reduce the risk of hip fracture. In the United States, DXA testing to measure a BMD T-score is well-established for identifying patients who will most benefit from such treatment. However, contributing to what some call a crisis in osteoporosis care,^3^ DXA has two limitations that inhibit further prevention of hip fracture: most individuals who should be tested diagnostically with DXA are not tested^4^; and for those who are tested, DXA has only modest sensitivity for predicting hip fracture.^5^ As a result, most individuals who would benefit from drug treatment never get treated. Clinical adoption of alternative diagnostic tests that overcome those limitations could help improve fracture prevention compared to relying exclusively on DXA testing.

Biomechanical computed tomography (BCT) analysis is one such potential alternative. It is well established that hip fracture is more strongly associated with femoral strength by finite element analysis of a hip-containing CT scan than with BMD.^6^ The VirtuOst BCT test provides such a measurement for femoral strength, as well as DXA-equivalent BMD T-scores at the hip.^7^ Indicated by the FDA in the United States for identifying osteoporosis, assessing fracture risk, and monitoring therapy, this test can repurpose a CT scan from a patient’s other medical care (“opportunistic” use), obviating additional imaging or radiation exposure. Biomechanically, an individual will fracture their hip upon a fall if the resulting impact force exceeds the breaking strength of their proximal femur.^8–10^ A BCT-type test for femoral strength that also accounts in some way for a patient’s fall risk and impact force should theoretically further improve fracture prediction over strength or BMD alone. In this study, we introduce the next generation of the VirtuOst BCT test that accounts for the three major determinants of hip fracture (fall risk, impact force, and femoral strength) by providing an integrative “BCT Risk Score” for predicting hip fracture. The approach is designed to only require input from a typical abdominal-pelvic CT scan, taken for any medical indication. We also demonstrate that, in a large and diverse clinical setting, such a multi-faceted biomechanical analysis improved prediction of hip fracture compared to both the hip BMD T-score and the Fracture Risk Assessment Tool (FRAX, Centre for Metabolic Bone Diseases, University of Sheffield).^11^

## Materials and methods

### Study design

This analysis utilized data from a case-cohort observational study of a first incident hip fracture, in which BCT testing at the time of the CT scan (start of observation) was the exposure. The BCT measurements were supplemented by data obtained from the electronic medical record. The analysis was part of the ongoing Fracture, Osteoporosis, and CT Utilization Study (FOCUS), originally reported in 2018^12^ and now expanded. The study was approved by the Kaiser Permanente Southern California (KPSC) Institutional Review Board with a waiver of informed consent.

### Participants

The underlying source population of 341364 patients were members of KPSC, a large integrated healthcare organization, who were aged ≥65 yr and had any type of abdominal-pelvic CT scan taken during their clinical care between January 1, 2005 and July 1, 2018, at 1 of 17 different medical centers and 11 associated imaging facilities. Patients were excluded if they had a hip fracture before the CT scan, any bone pathology that would exclude them from osteoporosis care, a hip implant, or had missing or otherwise unusable CT scans (Figure S1). From the remaining 271 389 patients, an initial case-cohort sample of 11 461 patients was developed (7913 women, 3548 men) that was comprised of: (1) all hip fracture cases from the population (within 10 yr after the patient’s CT scan) with analyzable CT scans for BCT analysis and complete medical record data and (2) a randomly selected sample of the underlying source population (“the random sample”, 5978 patients) that was age-matched between the sexes, had approximately the same number of patients per sex as all the hip fracture cases, and that included any hip fracture cases that occurred in that sample (123 cases in 4149 women; 31 cases in 1829 men).

The initial case-cohort sample was split into two independent groups based on the geographic locations of the medical centers and imaging facilities: a “development” set and a “validation” set (Figure S2). For the development set, patients were selected from the western part of the greater Los Angeles area, which included 12 medical/imaging facilities (52-1044 patients per facility; 6692 patients total). Data from only these patients were used to develop the new BCT Risk Score algorithm, which was then locked. The validation set comprised of patients in all southern California regions beyond the western greater Los Angeles area, which included 16 medical/imaging facilities (10-1084 patients per facility, *N* = 4769 patients total); only their data were used to validate the (locked) algorithm.

In defining both the development and validation sets, some patients from the initial case-cohort sample were excluded, recoded, or dropped out (Figure S2). Patients were excluded if they took ≥180 d of an osteoporosis medication during the observation period, based on prescription fill data. The rationale was that BCT is typically used clinically to assess fracture risk for an untreated patient. Because a 5-yr predictive time frame was considered of primary clinical interest, we defined our primary clinical outcome as a hip fracture within 5 yr of the CT scan. Any patient with a hip fracture more than 5 yr after the CT scan was either recoded to a no-fracture control or excluded depending on whether they were included or not, respectively, in the random sample. Any non-fracturing patient in the random sample who did not survive follow-up to 5 yr was dropped. These dropout patients either died or left the KPSC system—we could not distinguish due to IRB constraints—or entered the study within 5 yr of the observation end date (June 30, 2020). For the development set only, patients were also excluded if they did not have a complete set of accurate measurements for all physical traits needed for the BCT Risk Score. To mimic clinical care, any such missing measurements in the validation set (typically due to truncation of the relevant anatomy by a small field of view in the CT scan) were imputed (for 16 patients), using the sex- and age-based regressions of those measurements from the random sample. The final analysis sample comprised of 3017 patients (1781 with hip fracture; 1236 without hip fracture) for the development set and 2124 patients (1293 with hip fracture; 831 without hip fracture) for the validation set.

### Measurements

*Hip BMD T-score.* Because DXA measurements were not available for all patients and the study design was built around CT scans, the FDA-cleared and DXA-equivalent (see Supplementary Materials for details) hip BMD T-score^13^ (lower of the FN and TH regions) was measured from the CT scans, using VirtuOst (version 2.4, O.N. Diagnostics).

*FRAX hip score.* ^11^ Using the online FRAX calculator for the United States, we calculated the race-specific 10-yr absolute risk of hip fracture. For input (see Supplementary Materials for details), we used the VirtuOst-measured and DXA-equivalent BMD T-score at the FN (female White young reference for both sexes) and included all risk factors (from the patient’s medical record) except for parental fracture, which was not reliably available.

*The BCT Risk Score.* Add-on software (VirtuOst BCT Plus) to VirtuOst was developed to calculate the BCT Risk Score, which is a metric of hip fracture risk ranging from 0 to 100. The algorithm for the BCT Risk Score uses the femoral strength measurement from VirtuOst and adds age (always available in the metadata of a clinical CT scan) and various CT-based measurements of physical traits that, as a group, relate to all three biomechanical determinants of hip fracture: muscle characteristics that may be associated with falling or poor balance^14^; subcutaneous fat at the hip that can cushion the impact of a fall^10^^,^^14^^,^^15^; skeletal size, which also relates to impact forces during a fall^8^^,^^16^; and additional bone measurements that might relate to bone quality elements not reflected in the VirtuOst measurement of femoral strength. Neither weight, height, nor any clinical factors were considered because the test was designed for clinical logistical purposes to only require information obtainable directly from the CT scan.

Candidate physical traits were selected to maximize the area under the receiver-operator curve (AUC) for predicting hip fracture within 5 yr of the CT scan for the development set. The following criteria were used for trait selection:

- has a plausible biomechanical role in risk of hip fracture;
- is well defined and can be reliably obtained from a typical clinical abdominal-pelvic CT scan;
- is weakly correlated (R^2^ ≤ 0.25) with age and femoral strength in the random sample;
- predicts (*p* < .0001) hip fracture when used alone as a predictor; and
- predicts (*p* < .0001) hip fracture when used with all of the other BCT Risk Score traits.

**Figure 1:**
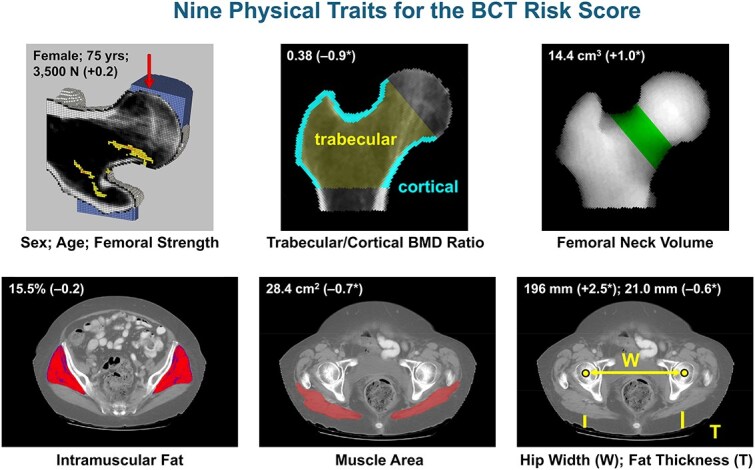
The nine physical traits used to calculate the BCT Risk Score. See Table 1 for a description of each physical trait. This particular patient was a 75-yr-old woman who had a BCT Risk Score of 91 (on a scale of 0-100). Her hip BMD T-score was −1.9 (osteopenia) and she fractured her hip 3 yr after the CT scan. Values of each measured physical trait for the patient are shown above, with Z-scores in parentheses; any starred value for a Z-score indicates that, compared to having an age- and sex-matched mean value of that trait (Z = 0.0), that trait is increasing her risk of hip fracture. Thus, in addition to her advanced age, this patient is at high risk of hip fracture because of low trabecular/cortical BMD ratio, large skeletal size (high FN volume, wide hips), small muscle size, and low fat thickness.

This process identified age, femoral strength, and six other physical traits (Figure 1, Table 1) as risk factors: trabecular/cortical BMD ratio, subcutaneous fat thickness posterior to the femoral head, muscle area of the gluteus maximus—higher values of these variables reduce the risk of fracture; and intramuscular fat content (% volume) of the gluteus medius/minimus, FN volume, and hip width—higher values of these variables increase the risk of fracture. Sex was not significant (*p* = .77) nor were there any strong sex interactions, so no sex terms were included. For this model, the AUC for the pooled sexes was 0.89 (3017 patients in the development set). This AUC was higher than when using strength and age together (AUC = 0.83), confirming that adding these six other physical traits did improve the prediction. Accounting for variations in some of the main imaging-related parameters did not affect the AUC (see Supplementary Materials) and thus imaging parameters were not included in the model formulation.

**Table 1:** Details of the nine physical traits used to calculate the BCT Risk Score. Increasing values of each trait can either increase or decrease the BCT Risk Score, as shown. R^2^ values are shown (when *p* < .05) for the univariate relation between age and femoral strength with each of the physical traits, for the random sample of patients from the underlying population^a^ (*N* = 3965 women; *N* = 1774 men).

**Physical trait**	**Description**	**Increases/decreases** **risk**	**R** ^**2**^ **vs age**			**R** ^**2**^ **vs strength**		
			**Female**	**Male**	**Pooled**	**Female**	**Male**	**Pooled**
**Sex**	Patient’s sex							
**Age (years)**	Patient’s age at the time of CT exam	Increases	—	—	—	0.091	0.044	0.050
**Femoral strength (N)**	Breaking strength of the proximal femur for a simulated sideways fall; obtained from the *VirtuOst BCT* analysis (left side, nominally)	Decreases	0.091	0.044	0.050	—	—	—
**Trabecular/cortical BMD ratio**	Ratio of trabecular to cortical BMD in the proximal femur. “Cortical” defined as all bone tissue with apparent density ≥ 1.0 g/cm^3^ or within 2 mm of the periosteal surface; see Figure 1 for region of interest	Decreases	0.045	0.021	0.037	0.161	0.133	0.090
**Fat thickness (mm)**	Thickness of the subcutaneous fat, posteriorly and directly below the femoral head center, as measured from a 10-mm-thick transverse section through the two femoral head centers. Lower value of left/right sides	Decreases	0.030	0.008	0.021	0.019	0.004	0.013
**Muscle area (cm** ^**2**^ **)**	Cross-sectional area of the gluteus maximus in a 10-mm-thick transverse section through both femoral head centers. Lower value of left/right sides	Decreases	0.100	0.115	0.086	0.131	0.137	0.225
**Intramuscular fat (%)**	Percent fat tissue (by volume) in the gluteus medius and minimus muscles, in a 10-mm-thick transverse section centered 1.5 femoral head-diameters proximal to the head centers. Average value of left/right sides	Increases	0.124	0.105	0.117	0.073	0.053	0.060
**Femoral neck volume (cm** ^**3**^ **)**	Volume of the FN, for the same region of interest as used to measure the FN BMD T-score in the *VirtuOst BCT* analysis	Increases	0.010	0.014	0.008	0.010	0.005	0.150
**Hip width (mm)**	Three-dimensional distance between left and right femoral head centers	Increases	NS	0.004	NS	0.011	0.010	0.014

a For this regression analysis, because patients aged 90 and older were all coded with age = 90 (due to IRB constraints), these patients were omitted, as were all patients (*N* = 41) with any imputed values of the physical traits.

NS, not statistically significant (*p* > .05).

To measure these traits, each patient’s CT scan was resampled to a uniform 1 mm voxel resolution, the femoral strength measurement and scan calibration for BMD measurement were performed using VirtuOst,^7^ and then the other traits were measured using VirtuOst BCT Plus (Table 1). Some measurement details are noteworthy. For the trabecular/cortical BMD ratio, a unique region of interest—from just below the femoral head center to just below the greater trochanter—was defined to enable measurement even when the anatomical coverage of the proximal femur in the CT scan was limited. The “cortical” bone was defined as all bone within the outer 2 mm of the periosteal surface, as well as any bone with an apparent density ≥1.0 g/cm^3^. That approach averages any thin cortex with any adjacent trabecular bone in the “cortical” measurement, which can reduce the dependency of the trabecular/cortical BMD ratio on scanning parameters. The volume of the FN is for the same bone as used to measure the DXA-equivalent FN BMD. A U-net convolutional neural network was used to segment the gluteal muscles from images of two parallel 10-mm-thick transverse sections. To help correct for any patient misalignment, the transverse section for the gluteus maximus area measurement is rotated about an axis perpendicular to the frontal plane so it includes both femoral head centers. Using the same section, the surfaces of the subcutaneous fat (between muscle and skin) are identified directly posterior to the femoral head centers and the posterior fat thickness is measured. We measured the subcutaneous fat posteriorly rather than laterally because many patients can have truncated images laterally, either due to a large body size or a small field of view. The section for measuring the gluteus medius and minimus fat content is parallel to the maximus section but spaced superiorly by one femoral head diameter. To help account for variations in CT scanner and acquisition characteristics when measuring intramuscular fat, a generic effective energy of the CT scan (50 keV) is assumed and effects of intravenous contrast are corrected for. Then, based on known attenuation properties of pure muscle and pure fat at that energy, a biphasic mixture model of muscle and fat is assumed to calculate a volume fraction of fat in each voxel that is consistent energy-wise with the measured Hounsfield Unit of that voxel (Supplementary Materials); this is then averaged over all voxels within the segmented muscle to compute an average value of intramuscular fat content. The VirtuOst BCT Plus software automatically measures the six new traits; a technician then reviews all outputs and, if needed, adjusts any measurements (about 10% of scans required an adjustment, mostly minor); the BCT Risk Score was successfully measured for all patients who had a femoral strength measurement. Inter-operator repeatability analysis (Supplementary Materials) confirmed the robustness of these measurements.

The final step in developing the BCT Score was to adjust for sex-effects in how the underlying source population was sampled. Our case-cohort sample in FOCUS was initially chosen to have the same ratio (1:1) of fractures to no-fracture controls for both sexes, whereas in the general population that ratio is higher for women than men. Since FRAX is well validated for assessing absolute risk in the population, we calibrated the BCT Risk Score against FRAX to enforce that, at our nominal threshold for identifying “high-risk” patients, the proportion of patients testing positive for high-risk status would be the same for BCT and FRAX (hip risk ≥3.0%, see Table 1), for each sex. To make this adjustment, a sex-specific constant was added to the logit before transforming it into a probability function. Mathematically, this is equivalent to randomly adding more no-fracture controls to the analysis sample, different numbers for the different sexes; we confirmed that adding such a constant does not affect the sensitivity, specificity, or AUC of the underlying predictive model. In this way, for each sex the logit was adjusted so that a binary threshold value of 75 (on a scale of 0-100) provided the same high-risk prevalence as FRAX.

### Statistical analysis

To validate the algorithm, only data from the validation set were used. The primary outcome was a first incident hip fracture within 5 yr of the CT scan, identified via ICD diagnosis codes for inpatient hospitalizations. In our main statistical analysis, for the same patients a sex-specific logistic univariate regression was used to calculate the AUC separately for the hip BMD T-score, the FRAX hip score, and the BCT Risk Score, which were then compared using DeLong’s test.^17^ Since each of these measurements in clinical care would be used alone to identify high-risk patients, no other covariates were included in these models. Using binary thresholds for identifying high-risk patients (hip BMD T-score ≤ −2.5; FRAX hip ≥ 3.0%; BCT Risk Score ≥ 75), sensitivity and specificity were calculated for predicting hip fracture within 5 yr. Confidence intervals were based on the normal approximation to the binomial distribution. The diagnostic odds ratio was also calculated, which is the odds of hip fracture for testing positive divided by the odds of hip fracture for testing negative. Beyond this main analysis, a multivariable model was run that included the (continuous) BCT Risk Score and the following clinical risk factors: weight, height, age, race/ethnicity (White, Asian, Black, or Hispanic), and (all binary): smoking history, rheumatoid arthritis, Alzheimer’s, secondary osteoporosis, alcohol abuse, use of glucocorticoids, and major fracture in previous year. As a secondary outcome and relevant to “imminent” fracture risk, we repeated the main analysis using hip fracture at 2 yr as the outcome, with appropriate recoding for fracture and no-fracture/dropout status.

Various ancillary analyses (Supplementary Materials) were performed to confirm robustness of the main analysis, including stratified analyses on patients with osteopenia, without osteoporosis, age 70 or older; removing the exclusion criteria for drug treatment during observation and dropouts; and comparing performance for the 220 patients (sex-pooled) who had DXA within 100 d of the CT scan, using both the DXA and BCT measurements of the hip BMD T-score. An additional ancillary analysis investigated effects of the main imaging parameters (intravenous contrast, scan slice thickness, and scan reconstruction orientation) on the overall prediction.

To assess clinical effectiveness, we calculated the positive predictive value (PPV) and estimated a number of preventable hip fractures over a 5-yr period, by sex and for ages ≥65 and ≥70. Positive predictive value is the risk of fracturing within the next 5 yr after the CT scan, having tested positive for being high-risk. This metric can therefore be used to quantitatively assess that, as a group, patients who test positive for being high-risk (or very-high-risk) by the BCT Risk Score are indeed at high risk (or very high risk) of fracture. The estimated number of preventable hip fractures (per 1000 tested patients) addresses potential clinical impact. These metrics were calculated as follows: 


\begin{align*} &\mathrm{PPV}=\mathrm{S}\times \mathrm{PopFR}\ /\ \mathrm{TestPR}\\&\mathrm{Preventable}\ \mathrm{hip}\ \mathrm{fractures}\ \left(\mathrm{per}\ 1000\ \mathrm{tested}\ \mathrm{patients}\right)\\&\qquad=\mathrm{S}\times \mathrm{PopFR}\times \mathrm{E}\times 1000 \end{align*}

where PopFR is the population fracture risk, which was measured from the random sample for the untreated patients (ie, fracture risk for patients who remain untreated) and excluded drop-outs; TestPR is test prevalence rate, which is the proportion of patients who test positive for high-risk in the random sample at baseline and thus includes any future dropouts, reflecting clinical care in which the future status of any tested patient is unknown; S is the test sensitivity, which was obtained from the validation set (which excluded treated patients); and E = the treatment efficacy, assumed to represent alendronate (risk reduction = 0.53^18^). To estimate the number of preventable fractures, we assumed all high-risk patients are treated and on average have a reduced risk of hip fracture as per the assumed treatment efficacy. These analyses were done for a range of BCT threshold values (75-95, in 5-point increments). The PPV at the FRAX thresholds of 3.0% and 4.5% provided a quantitative reference as to what level of risk is acceptable clinically for defining high-risk and very-high-risk status, respectively.^19^^,^^20^

To help interpret how each risk factor in the model contributes to a patient’s increased BCT Risk Score, a sex-specific Z-score (race/ethnicity pooled) was calculated for each of the physical traits. Using the data for the random sample, linear regressions vs age were constructed by sex for each of the physical traits. From those regressions, a patient’s Z-score for each trait was defined as the difference between the patient’s value and the sex-specific age-predicted mean value for that trait, divided by the standard error of the age-regression for that trait. To assess how the traits differed between patients classified as high-risk vs not-high-risk (BCT Risk Score < 75), for the patients in the random sample the age-adjusted mean values of each trait were compared between high-risk vs not-high-risk status and between the sexes. All statistics were performed in JMP Pro version 17 (JMP Statistical Discovery, LLC).

## Results

For patients in the random sample, the mean age was 72 yr for both sexes, with slightly over 60% being age 70 or older and almost 50% being non-White (Table 2). Women had a higher prevalence of obesity, rheumatoid arthritis, glucocorticoid use, and previous major fracture than the men, whereas men had a higher prevalence of diabetes, smoking, and alcohol abuse. Women had an almost two-fold (*p* < .0001) higher prevalence of high-risk classification than men in terms of either hip BMD T-score (18.0% vs 10.3%), FRAX hip fracture probability (34.6% vs 20.0%), or femoral bone strength (30.7% vs 16.1%). By design, prevalence for high-risk by FRAX was the same as for the nominal high-risk threshold (≥75) for the BCT Risk Score.

**Table 2:** Characteristics of the randomly selected sample of the underlying source population. Mean ± SD for continuous variables, unless noted; percentages for categorical variables. The sample was age-matched between the sexes; total number per sex approximately matches the female:male ratio of hip fractures in the underlying source population.

**Characteristic**	**Women**	**Men**	***p*-value** ^a^
**Number of patients**	4149	1829	
**Age (years)** ^b^	72 (67-78.5)	72 (68-79)	.71
** Age range (years)** ^c^	65-90	65-90	
** Age ≥ 70 yr (%)**	62	61.2	.58
**Race/ethnicity**			.002
** Non-Hispanic White (%)**	52.5	56	
** Hispanic (%)**	26.5	24.8	
** Black (%)**	11.4	8.5	
** Asian or Pacific Islander (%)**	8.5	9.3	
** All other (%)**	1.1	1.5	
**Height (m)**	1.60 (0.07)	1.74 (0.08)	<.0001
**Weight (kg)**	70.9 (17.4)	83.7 (17.1)	<.0001
**Body mass index (kg/m^2^)** ^d^	27.8 (6.3)	27.6 (5.0)	.26
**Obese (BMI ≥ 30, %)** ^d^	30.4	26.3	.002
**Diabetes (%)**	26.3	29.9	.004
**Rheumatoid arthritis (%)**	39.3	27.6	<.0001
**Secondary osteoporosis (%)**	11.4	12.9	.098
**Glucocorticoid use (%)**	11.8	7.6	<.0001
**Smoker ≥1 yr (%)**	18.6	29.4	<.0001
**Alcohol abuse (%)**	1.4	4.5	<.0001
**Major fracture as adult (%)**	8.6	3.9	<.0001
**Hip BMD T-score**	−1.5 (1.1)	−0.7 (1.2)	<.0001
** High risk (%)**	18	6.2	<.0001
**Femoral strength (N)**	3460 (860)	4570 (1120)	<.0001
** High risk (%)**	30.7	16.1	<.0001
**FRAX 10-yr risk hip fracture (%)** ^b^	1.7 (0.7-4.2)	1.1 (0.5-2.5)	<.0001
** High risk (%)**	34.6	20	<.0001
**BCT Risk Score** ^b^	56 (29-84)	35 (15-67)	<.0001
** High risk (%)**	34.5	19.9	<.0001

a *p*-value compares both sexes: for continuous variables, *t*-test (two-tail) except Wilcoxon test if not normally distributed; for categorical, Pearson’s test.

b Not normally distributed, median and interquartile range.

c All ages above 90 yr = 90 to protect patient identity (IRB constraints).

d BMI data were not available for 26 women and 15 men.

BMD T-score is lower of FN and TH regions, using the young-female White reference from NHANES for both sexes. FRAX uses the FN BMD T-score and had input for all risk factors except parental history of fracture. “High Risk” defined as: hip BMD T-score ≤ −2.5, femoral strength ≤ 3000 N (women) or 3500 N (men), FRAX hip ≥ 3.0%, or BCT Risk Score ≥ 75.

**Figure 2:**
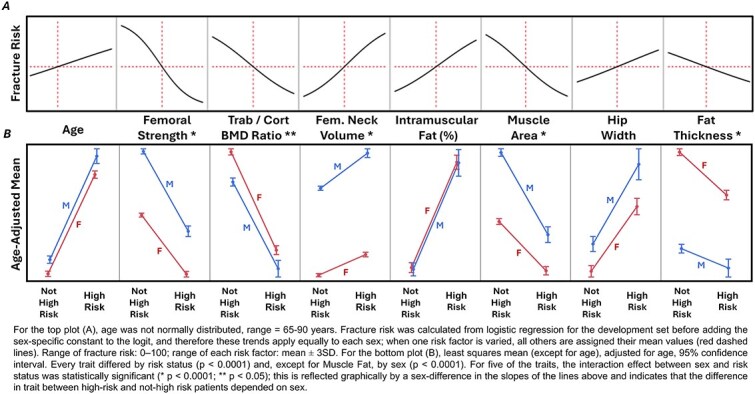
(A) Profile of the risk factors. General dependence of the calculated fracture risk on each risk factor (pooled sexes); the larger the vertical range, the greater the influence of that risk factor on the fracture risk. (B) Physical traits of the high-risk patient. Comparison of age and the age-adjusted physical traits between sex and risk status [high risk (BCT Risk Score ≥ 75) vs not high risk (BCT Risk Score < 75)] for all patients in the random sample of the underlying population (*N* = 4117 women and *N* = 1820 men, which excludes the 41 patients who had any imputed value for any trait measurement).

For patients in the development set, profiling of the risk factors for the BCT Risk Score showed that the sex-pooled calculated risk (before adjusting the logit with a sex-specific constant) was most influenced by femoral strength, FN volume, trabecular/cortical BMD ratio, intramuscular fat, and muscle area and less so by age, hip width, and posterior fat thickness (Figure 2A). For patients in the random sample, when stratified by sex and the BCT Risk Score to high-risk vs not-high-risk status, all age-adjusted physical traits differed significantly by high-risk status, and all traits differed between the sexes except for intramuscular fat (Figure 2B). For both sexes, high-risk patients had significantly higher age and age-adjusted femur neck size, hip width, and intramuscular fat, and significantly lower age-adjusted femur strength, trabecular/cortical BMD ratio, muscle area, and posterior fat thickness.

**Figure 3:**
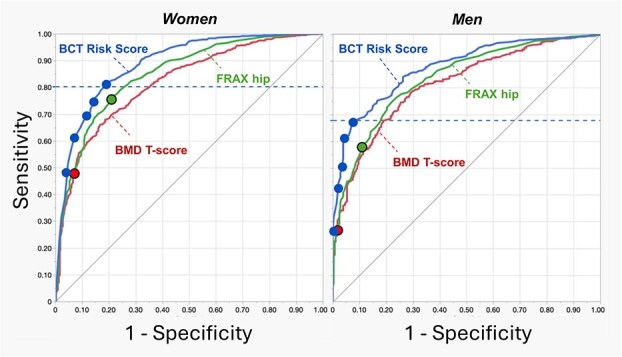
Receiver-operator-characteristic curves for predicting a future hip fracture within 5 yr of the CT scan, for all patients in the validation set (*N* = 1386 women; *N* = 738 men), comparing the hip BMD T-score (lower of TH and FN regions), FRAX hip (w/ BMD), and the BCT Risk Score. Solid points show sensitivity and specificity values at the binary thresholds (BMD T-score = −2.5; FRAX hip = 3.0%; BCT Risk Score = 75, 80, 85, 90, or 95, top to bottom, respectively); dashed horizontal line shows the optimal value of sensitivity for the BCT Risk Score based on maximizing the Youden index. See Table 3 for statistical comparisons.

For patients in the validation set, AUC values for predicting hip fracture indicated that the BCT Risk Score (as a continuous variable) had the best overall fracture prediction (*p* < .0001). For the main analysis, there were 1386 women (837 with hip fracture, 549 without) and 738 men (456 with hip fracture, 282 without). The AUC values were higher for the BCT Risk Score than the BMD T-Score and the FRAX score both for the women (0.89 vs 0.82 vs 0.85, respectively) and the men (0.88 vs 0.81 vs 0.84; Table 3). Those trends persisted in the secondary analysis for the 2-yr fracture outcome (Table 3). For both sexes, the receiver-operator-characteristic curves for predicting hip fracture at 5 yr (Figure 3) demonstrated clear separation between the BCT Risk Score vs the BMD T-Score and FRAX score, indicating that for almost all values of specificity, the corresponding sensitivity was highest for the BCT Risk Score. For the pooled sexes, AUC values for the BCT Risk Score were also similar when applied to patients in the development vs validation sets (0.89 vs 0.88, respectively) indicating that the algorithm for the BCT Risk Score was not overfit. For the validation set, excluding patients with any imputed values of the physical traits (13 women, 3 men) did not change AUC values; and for each sex adding in all available clinical risk factors to the (continuous) BCT Risk Score only increased the AUC by 0.007 units.

**Table 3:** Comparison of hip fracture prediction between BMD, FRAX, and BCT within 5 yr (primary outcome) and 2 yr (secondary outcome) of the CT scan, in women and men aged 65 and older. No patient had more than 180 d of any osteoporosis medication during the observation period. Parentheses denote 95% CIs.

**5-Year hip fracture**											
	AUC			Sensitivity (%)			Specificity (%)			DOR	
**Women (*N* = 837 FX; 549 No-FX)**											
**Hip BMD T-score**	0.818	[0.794-0.839]^a,b^		47.8	[47.7-47.8]		92.9	[92.9-92.9]		12.0	[8.4-17.0]
**FRAX hip**	0.849	[0.828-0.869]^a^		75.9	[75.8-75.9]		79.1	[79.0-79.1]		11.9	[9.2-15.4]
**BCT Risk Score**	0.887	[0.868-0.904]		81.4	[81.3-81.4]		80.0	[79.9-80.0]		17.4	[13.3-22.9]
**Men (*N* = 456 FX; 282 No-FX)**											
**Hip BMD T-score**	0.813	[0.780-0.842]^a,c^		26.8	[26.7-26.8]		98.6	[98.6-98.6]		25.4	[9.3-69.6]
**FRAX hip**	0.835	[0.804-0.862]^a^		58.1	[58.1-58.2]		89.4	[89.3-89.4]		11.7	[7.6-17.8]
**BCT Risk Score**	0.876	[0.850-0.898]		66.9	[66.8-66.9]		92.2	[92.2-92.2]		23.9	[14.8-38.5]
**2-Year hip fracture**											
	AUC			Sensitivity (%)			Specificity (%)			DOR	
**Women (*N* = 490 FX; 1084 No-FX)**											
**Hip BMD T-score**	0.801	[0.776-0.823]^a,b^		52.2	[52.2-52.3]		88.3	[88.3-88.3]		8.2	[6.4-10.7]
**FRAX hip**	0.835	[0.813-0.855]^a^		79.6	[79.6-79.6]		72.1	[72.1-72.2]		10.1	[7.8-13.1]
**BCT Risk Score**	0.875	[0.857-0.892]		85.3	[85.3-85.3]		73.0	[72.9-73.0]		15.7	[11.8-20.8]
**Men (*N* = 235 FX; 542 No-FX)**											
**Hip BMD T-score**	0.794	[0.755-0.828]^a,c^		30.6	[30.5-30.7]		97.1	[97.0-97.1]		14.5	[8.2-25.7]
**FRAX hip**	0.819	[0.784-0.849]^a^		59.2	[59.1-59.2]		84.7	[84.7-84.7]		8.0	[5.6-11.4]
**BCT Risk Score**	0.870	[0.840-0.895]		73.2	[73.1-73.2]		87.3	[87.2-87.3]		18.7	[12.8-27.5]

AUC = area under the receiver-operator curve by logistic regression for the predictor as a continuous variable; DOR = diagnostic odds ratio = odds of fracture if test positive/odds of fracture if test negative. Sensitivity and specificity and DOR use the following binary thresholds: BMD T-score ≤ −2.5; FRAX hip ≥ 3.0%; BCT Risk Score ≥ 75. BMD T-score at the hip uses the lower T-score at the FN and TH regions and the NHANES young-female (White) reference. FRAX hip is calculated (USA race-specific) using the FN BMD T-score (young-female White reference) as input as well as all clinical risk factors except parental history of fracture. N = sample size for the statistical analysis. FX = incident hip fracture; No-FX = no incident hip fracture, all dropouts excluded. Statistical comparisons made only for AUC: ^a^*p* < .0001 vs BCT Risk Score; ^b^*p* < .0001 and ^c^*p* < .05 vs FRAX; Chi-Square, DeLong’s method.

**Figure 4:**
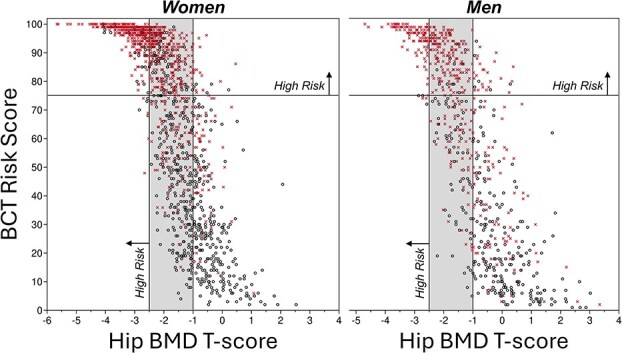
The relation between the BCT Risk Score (range 0-100) and hip BMD T-score for all patients in the validation set (*N* = 1386 women; *N* = 738 men). Thresholds are shown for identifying patients at high risk of a future hip fracture (BCT Risk Score ≥ 75; BMD T-score ≤ −2.5) and those with normal bone mass (BMD T-score ≥ −1.0) at the hip; the shaded region denotes patients with low bone mass (aka osteopenia). The NHANES white female young-reference was used for all T-score calculations. "x" points are patients who fractured their hip within 5 yr after the CT scan; "o" points are the no-fracture controls. See Table 3 for sensitivity, specificity, and diagnostic odds ratio for the different high-risk classifications.

Using the thresholds for binary classification, sensitivity for predicting hip fracture at 5 yr was highest for BCT compared to BMD and FRAX (Table 3). In absolute terms compared to the BMD T-score, the sensitivity for the BCT Risk Score in the women was higher by 33.6 points (81.4% vs 47.8%) and specificity lower by 12.9 points, a net gain of sensitivity vs specificity of 20.7 points; in the men the sensitivity higher by 40.1 points (66.9% vs 26.8%) and specificity lower by 6.4 points, a net gain of 33.7 points. Scatter plots of the BCT Risk Score vs hip BMD T-score illustrate how the high-risk patients were related between both tests (Figure 4). Both sensitivity and specificity were consistently higher for BCT than FRAX, for both sexes. For the women, the diagnostic odds ratio—the relative risk of fracture for patients testing positive vs negative—was similar between the hip BMD T-score and FRAX and trended higher for BCT (BMD 12.0, FRAX 11.9, BCT 17.4); for the men, the diagnostic odds ratio was similar between the hip BMD T-score and BCT and trended lower for FRAX (BMD 25.4, FRAX 11.7, BCT 23.9). Overall, these results were robust to multiple modeling parameters and cohort stratifications, including patients with osteopenia, without osteoporosis, those age 70 and older, and including those on drug treatment and all dropouts (Table S3). Results were also robust when patients were stratified by the main imaging parameters (Table S2). In the secondary analysis for the 2-yr fracture outcome, all trends in the main analysis persisted (Table 3); for each test, sensitivity was higher for the 2-yr than 5-yr outcome, reaching 85.3% in women and 73.2% in men for the BCT Risk Score.

Using FRAX as the clinical reference, the clinical effectiveness metrics (Table 4) indicated that, for women age 65 and older and men age 70 and older, the nominal BCT high risk threshold of 75 identified both women and men who were at a clinically acceptable level of high-risk of hip fracture at 5 yr; a BCT threshold of 85 for women and 90 for men identified those at a clinically acceptable level of very high risk. For identifying high-risk patients, PPV was always at least as high as for the hip BMD T-score (≤−2.5) and the BCT Risk Score (≥75) as for FRAX (≥3.0%), our assumed standard for defining high-risk status. Reflecting the results for test sensitivity, the estimated number of preventable hip fractures per 1000 tested women age ≥65 was highest for the BCT Risk Score, being 67% higher than for the hip BMD T-score (25 vs 15) and slightly higher than for FRAX (25 vs 23); larger relative improvements occurred for the men. Using FRAX ≥4.5% as a clinical standard for defining very-high-risk status, its level of risk (by PPV) was matched or exceeded by the BCT thresholds of 85 and above for women aged 65 and older and by thresholds of 90 and above for men aged 70 and older.

**Table 4:** Clinical performance characteristics for predicting hip fracture within 5 yr: proportion of patients testing positive, positive predictive value (PPV), and number of preventable hip fractures (within 5 yr per 1000 tested patients). For each patient category, FRAX (shaded rows) is considered the clinical reference for quantifying an acceptable level of risk (lowest acceptable PPV) for high-risk and very-high-risk (**bolded**) clinical classifications. Multiple threshold values are shown for the BCT Risk Score.

**Patient category and test type**	**Positive test rate (%)**	**Sensitivity for hip fracture (%)**	**PPV for hip fracture (%)**	**High risk or very high risk**	**Preventable hip fractures per 1000 tests**
**Women (age ≥ 65)**					
***Population risk = 5.8%***					
**High risk (FRAX ≥ 3.0%)**	34.6	75.9	12.7	High Risk	23
**Very high risk (FRAX ≥ 4.5%)**	23.5	62.7	15.4	**Very High Risk**	19
** Hip BMD T-score ≤−2.5**	18.0	47.8	15.3	High Risk	15
** BCT Risk Score ≥75**	34.5	81.4	13.6	High Risk	25
** BCT Risk Score ≥80**	29.8	76.3	14.8	High Risk	23
** BCT Risk Score ≥85**	24.4	70.4	16.6	**Very High Risk**	22
** BCT Risk Score ≥90**	18.5	62.1	19.4	**Very High Risk**	19
** BCT Risk Score ≥95**	12.4	48.2	22.4	**Very High Risk**	15
**Women (age ≥ 70)**					
***Population risk = 8.3%***					
**High risk (FRAX ≥ 3.0%)**	48.9	82.8	14.1	High Risk	36
**Very high risk (FRAX ≥ 4.5%)**	34.3	69.6	16.9	**Very High Risk**	31
** Hip BMD T-score ≤−2.5**	24.2	51.7	17.8	**Very High Risk**	23
** BCT Risk Score ≥75**	48.8	86.4	14.7	High Risk	38
** BCT Risk Score ≥80**	43.0	81.7	15.8	High Risk	36
** BCT Risk Score ≥85**	36.0	76.8	17.7	**Very High Risk**	34
** BCT Risk Score ≥90**	27.8	68.2	20.4	**Very High Risk**	30
** BCT Risk Score ≥95**	18.9	53.4	23.5	**Very High Risk**	24
**Men (age ≥ 65)**					
***Population risk = 3.4%***					
**High risk (FRAX ≥ 3.0%)**	20.0	58.1	9.8	High Risk	10
**Very high risk (FRAX ≥ 4.5%)**	9.7	38.8	13.4	**Very High Risk**	7
** Hip BMD T-score ≤−2.5**	6.2	26.8	14.5	**Very High Risk**	5
** BCT Risk Score ≥75**	19.9	66.9	11.3	High Risk	12
** BCT Risk Score ≥80**	16.9	61.6	12.2	High Risk	11
** BCT Risk Score ≥85**	13.8	51.3	12.5	High Risk	9
** BCT Risk Score ≥90**	10.3	42.5	13.9	**Very High Risk**	8
** BCT Risk Score ≥95**	5.7	26.5	15.6	**Very High Risk**	5
**Men (age ≥ 70)**					
***Population Risk = 4.5%***					
**High Risk (FRAX ≥3.0%)**	30.0	65.0	9.8	High Risk	16
**Very High Risk (FRAX ≥4.5%)**	14.6	43.5	13.5	**Very High Risk**	10
** Hip BMD T-score ≤−2.5**	8.8	28.5	14.8	**Very High Risk**	7
** BCT Risk Score ≥75**	29.7	73.1	11.2	High Risk	18
** BCT Risk Score ≥80**	25.4	68.2	12.2	High Risk	16
** BCT Risk Score ≥85**	21.1	57.9	12.5	High Risk	14
** BCT Risk Score ≥90**	16.1	48.6	13.7	**Very High Risk**	12
** BCT Risk Score ≥95**	8.8	38.4	19.7	**Very High Risk**	9

Population Risk, the same for each test within each patient group, is for a future hip fracture within 5 yr and is based on observed fractures in the random sample for untreated patients and excluding dropouts. A value of 5.8% means that for 1000 patients there should be 58 hip fractures within the next 5 yr. See text for details of the calculations.

## Discussion

These results from a large and diverse clinical care setting demonstrate the superiority of the BCT Risk Score over the hip BMD T-score as a diagnostic test for predicting a future hip fracture, with improved performance also compared to FRAX (albeit without information on parental fracture, and not including “FRAXplus”). Compared to both BMD and FRAX, the AUC for prediction for the (continuous) BCT Risk Score was the highest, indicating improved overall fracture prediction. The nominal binary threshold (≥75) to identify high-risk patients, which did not use any fracture-outcome data for its calibration, was shown to be effective in terms of maximizing test sensitivity for identifying patients who subsequently fractured. Use of this threshold should therefore improve fracture prevention. Those results were robust to sex and to different patient categories, for example, those with osteopenia, without osteoporosis, and over age 70 (Table S3) and applied also to our two-year “imminent risk” secondary outcome. Our analysis of PPV used FRAX as the reference for defining acceptable risk levels for classifying high-risk and very-high-risk patients. That analysis provided a quantitative basis for validating the various BCT threshold values for these classifications. Based on that validation of the BCT Risk Score algorithm, as per clinical guidelines^19^^,^^20^ those classified at high-risk by BCT can be considered for osteoporosis drug treatment and those at very-high-risk can be considered for first-line anabolic treatment. We conclude that, compared to both BMD and FRAX, the integrative BCT test better predicted hip fracture and its high sensitivity should improve fracture prevention.

These results suggest important potential clinical impact for this next-generation BCT test. BCT’s high sensitivity is clinically significant because it means that BCT should correctly identify substantially more hip-fracturing patients than BMD and more too than FRAX—these true-positives are the patients who will potentially benefit from osteoporosis drug treatment. By matching the prevalence rate of testing positive against the FRAX hip threshold (≥3.0%), the nominal high-risk threshold (≥75) for the BCT Risk Score was designed to avoid over-treatment, at least in countries that incorporate FRAX into treatment decision algorithms.^19^ Economically, the substantially higher sensitivity for BCT will make it cost-saving compared to standard-of-care DXA testing.^21^ Clinically, a BCT Risk Score could be interpreted to re-classify risk of patients without BMD-defined osteoporosis in a similar way to how the FRAX score is used, thus requiring minimal training of medical staff or adjustment of clinical guidelines. Logistically, as with such widely used tests as Cologuard, Heartflow FFR_CT_, or iRhythm Zio patch that all use a centralized facility for all tests, a patient’s recent CT scan is transmitted electronically to the O.N. Diagnostics facility for analysis and results are typically returned within 1-2 d of receiving the scan. This model ensures consistent expertise and quality control for all tests across the nation and is highly scalable in terms of volume. The main clinical use-case for the BCT Risk Score is the older patient without a recent DXA who is undergoing an abdominal-pelvic CT as part of their care, of which there are millions each year in the United States. Compared to undergoing a new DXA, BCT for these patients would be safer (no new X-ray exposure), more convenient and accessible (no new imaging procedure needed), and more effective (higher sensitivity and PPV). With appropriate physician awareness, it is not infeasible that 1-2 million such BCT tests could be performed each year in such patients. Our data on the number of preventable hip fractures suggest that doing so could prevent tens of thousands of hip fractures, which would save payors hundreds of millions of dollars annually compared to current standard of care. The main challenge for this scenario is not the availability of good diagnostic tests or medications—these all now exist. Instead, as with colorectal cancer screening, practice guidelines for osteoporosis screening should be expanded to recognize and utilize other safe and effective diagnostic bone density tests beyond DXA, such as BCT; and providers need to be available to treat the positive-testing patients.

This study has multiple attributes supporting its validity. The underlying patient population was large and diverse, comprising both sexes aged 65 and older, almost 50% non-White participants. Thus, the underlying regression model reflected substantial variations in anatomic morphology related to sex differences and variations in patient demographics. Supporting robustness of the measurements, there was a wide variation in the CT scanners and scanning protocols (Table S1), which did not compromise the overall prediction (Table S2). This robustness is likely because the variation in morphology across patients is large compared to any measurement errors introduced by variations in scanning parameters. In addition, the measurement repeatability (Supplementary Materials) was excellent. For calculating the PPV, the large number of fracture cases provided statistically robust estimates of test sensitivity, with tight CIs; and we based the prevalence rate (for testing positive) on all patients in the random cohort, mimicking clinical care in which the future status of the patient is unknown and thus avoiding issues with dropouts during observation. Supporting our estimates of PPV, the PPV for FRAX (≥3.0%) in women age 65 and older in our cohort, at 12.7%, is consistent with values of 12%-13% in women age 65 and older in the Study of Osteoporotic Fractures.^22^ Interpretation of the PPV data—the risk of fracturing within five years if testing positive—was also aided by using FRAX as a clinical reference, enabling us to quantitatively assess the risk levels for the different BCT threshold values. Statistically, the use of traditional logistic regression vs some type of machine learning reduced dependency of the model on cohort effects and the observed similarity of the AUC values for the development vs validation sets confirmed the model was not over-fit. The huge size of the source population (*N* = 341364 patients) enabled us to select an independent validation set that was geographically separate from the development set while including a large number of hip fracture cases in each set, and the various ancillary analyses (Tables S2 and S3) further supported robustness. The comparison between the BCT Risk Score and the BMD T-score and FRAX were based on repeated-measure analyses for the very same patients and from the same CT scans, adding robustness to the study design and statistical comparisons.

Generality of our findings beyond this large diverse cohort is supported by the mechanistic nature of the BCT Risk Score. All the physical risk factors included in the model are related to at least one mechanistic determinant of hip fracture—fall risk, impact force, or femoral strength—and all were highly statistically significant. Because the basic mechanisms of hip fracture do not depend on geographic location, it is unlikely that such a validated mechanistic approach would only outperform BMD in specific geographic locations. That said, studies in additional cohorts would confirm improved prediction over BMD and FRAX elsewhere; additional research is also needed to assess performance in various subgroups (eg, race/ethnicity or medical conditions). We caution that since the model was validated only in patients aged 65 and older, extrapolation to younger patients may not be valid. We also caution that, while we have demonstrated superior diagnostic performance, it remains to be demonstrated that clinical implementation of this improved diagnostic test does indeed improve fracture prevention.

Some caveats are noteworthy. First, we used BCT to measure the hip BMD T-score as opposed to using DXA. However, multiple studies have shown BCT and DXA are equivalent in terms of measuring the hip BMD T-score (see Supplementary Materials for details),^13^^,^^23–27^ predicting hip fracture,^12^^,^^23^ and identifying patients who will benefit from treatment.^28^ Our ancillary analysis on the subset of patients who had DXA within 100 d of the CT scan confirmed explicitly that the fracture prediction comparison vs BCT Risk Score did not depend on whether the BMD T-score was measured by DXA or BCT (Table S3). Although our FRAX measurement did not include a parental history of fracture, parameters studies (Table S4) showed that its omission should only have a minor effect on the overall fracture prediction by FRAX. Second, our source population all had an abdominal-pelvic CT, suggesting that this might represent a sicker population than the general older population. Substantial loss of muscle from any prolonged immobility, for example, might increase the BCT Risk Score more than reduce the BMD T-score. If such patients are at reduced risk of fracture due to their immobility, that might preferentially increase the number of false-positives for the BCT Risk Score. In that case, the performance reported here for the BCT Risk Score might underestimate its performance in a healthier more mobile population. However, this is a minor issue since the main use-case for the BCT Risk Score is for patients already with abdominal-pelvic CT. Third, given the retrospective nature of our study, we coded all no-fracture patients without the full 5 yr of follow-up as dropouts, which complicates interpretation of the specificity. For clinical effectiveness, the main issue for a diagnostic test beyond its sensitivity is not the specificity but the prevalence for testing positive, which we calculated from the random sample at baseline thus avoiding any issues related to dropouts. Fourth, although our novel method for measuring intramuscular fat (see Supplementary Materials) worked well for predicting hip fracture, it has not been directly validated with MRI or any gold standard. And finally, our Z-scores were pooled by race/ethnicity; having a breakdown by race/ethnicity could be useful clinically in order to better understand how an individual patient’s physical traits compare to population averages. Related, while variations in the main imaging parameters (presence of intravenous contrast, scan slice thickness, and reconstruction orientation) minimally affected the overall risk prediction, we did find (data not reported) that some of the physical traits were sensitive to some imaging parameters. The bone strength and BMD T-scores in VirtuOst are already adjusted for variations in scan slice thickness; and the intramuscular fat measurement is adjusted for intravenous contrast (see Supplemental Materials). Making additional adjustments to account for variations in imaging parameters, which may impact clinical decision-making at the level of the individual patient, remains a topic of future research.

This study represents an evolution in biomechanical approaches for improving clinical fracture prediction using a single image-based test and builds on a substantial body of prior work. Moving beyond BMD, multiple groups over the past two decades have reported on image-based estimates of the impact force and femoral strength for predicting hip fracture.^9^^,^^10^^,^^12^^,^^23^^,^^29–36^ Various measurements of muscle are associated with risk of hip fracture,^37–39^ presumably via an increased fall risk; and rheumatoid arthritis and diabetes—established clinical risk factors for fracture—are both associated with increased intramuscular fat.^40^^,^^41^ Independent of BMD, CT-based measurements of muscle at the hip^42^^,^^43^ and muscle and subcutaneous adipose tissue at the spine^44^ are associated with hip fracture. Our study is the first to show that integrating all three determinants of hip fracture in a patient-specific manner, and utilizing only existing clinical CT scans taken for any purpose, can increase sensitivity substantially compared to using only the hip BMD T-score.

In addition to improving fracture prediction, our results provide some new insight into hip fracture etiology. First, all the age-adjusted physical traits except intramuscular fat differed between the sexes (Figure 2B), emphasizing that the high-risk phenotype differs between the sexes. In the risk profile (Figure 2A), femoral strength, FN volume, intramuscular fat, muscle area, and trabecular/cortical BMD ratio were the more important risk factors in the model; age had about the same impact as posterior fat thickness. As expected, compared to women, we found that men had stronger bones, bigger bones, bigger muscles, and lower posterior fat thickness. Less obvious was that the differences in those physical traits between high-risk vs not-high-risk status also depended on sex (eg, for femoral strength, trabecular/cortical BMD ratio, FN volume, muscle area, and posterior fat thickness). Also less obvious was that, compared to women, men had a lower trabecular/cortical BMD ratio, wider hips, and the same intramuscular fat. Some of these sex differences put women at higher risk of fracture than men; others put them at lower risk. The complexity of these trends illustrates the clinical need for an integrative approach that simultaneously accounts for the three main determinants of fracture risk.

Second, the predictive role of the FN volume is noteworthy. It is known that a larger skeletal size increases the impact force upon a sideways fall.^16^ Rather than account for skeletal size via measurements of weight and height, which cannot be obtained from an abdominal-pelvic CT scan, we used two surrogate measures—hip width and FN volume. As part of our developmental work, we found that both were positively correlated with weight (R^2^ = 0.08 and 0.17, respectively) and height (R^2^ = 0.17 and 0.53). Our results also demonstrated that increasing values of each of these measurements, alone and when used in the multivariable model, increased the risk of hip fracture. Since larger bones are thought to be stronger, this result may seem counter-intuitive. However, we also found that the association between femoral strength (as computed by VirtuOst) and FN volume was weak (R^2^ ≤ 0.01 each sex, Table 1). This weak association reflects that bone density varies tremendously within each sex and that the underlying femoral strength biomechanics are complex.^45^ Thus, in our biomechanical framework, bone size primarily represents a surrogate for skeletal size and impact force.^46^ That said, recent work has shown evidence that larger FN width (as measured by DXA) is associated with greater loss of bone mass,^47^ suggesting that bone size may also represent a vector of future bone loss; additional work is needed to explore that concept.

It is also notable that low values of the trabecular/cortical BMD ratio, which was only weakly correlated with femoral strength (R^2^ < 0.20 each sex, Table 1), increased the risk of hip fracture. Mechanistically, we speculate that this measurement is a surrogate for the disruption of trabecular microarchitecture (not visible on a clinical CT scan) that can occur in highly porous trabecular bone, which can reduce overall structural redundancy and increase bone fragility.^48^ While risk of hip fracture was generally high in the oldest patients, many of the measured physical traits inherently embody effects of aging. Thus, as shown in Figure 2A, age as a variable per se only had a small influence in the prediction model. Further, in the development set, the AUC for predicting hip fracture was reduced by only 0.004 points when age was removed from the model. Mechanistically, the remaining small but statistically significant effect of the age variable likely serves as a surrogate for other biomechanically relevant age-related risk factors not reflected in the measured traits, such as deterioration in vision or neuromuscular function (eg, balance, reaction time) or the age-related loss of cortical bone ductility.^49^ The small effect on overall prediction (change in AUC of only 0.007 points) by adding multiple clinical risk factors suggests that little might be gained by integrating such factors into development of the (continuous) BCT Risk Score. However, further research is needed to investigate how using various clinical risk factors or FRAX might be combined with the binary BCT classifications to potentially improve risk stratification and clinical effectiveness.

Finally, in addition to better identifying patients at risk of a future hip fracture, the mechanistic and physical-trait-based BCT Risk Score may provide a new and objective basis for devising a patient-specific treatment plan that targets reversible physical traits for the patient that contribute to their increased risk of fracture. The Z-scores for the physical traits help identify which of a high-risk patient’s reversible traits contribute to their increased risk. For example, for high-risk patients, a negative Z-score for the trabecular/cortical BMD ratio might indicate an anabolic agent in order to build up the trabecular bone; a negative Z-score for the muscle area or a positive Z-score for the intramuscular fat might indicate physical therapy to improve muscle strength and balance; a negative Z-score for the posterior fat thickness might indicate a protective hip pad.^50^ For many patients, there may not be a single dominant deficient trait (see patient in Figure 1) and therefore risk might only be appreciably reduced by addressing all reversible deficiencies. Such a multi-faceted quantitative approach for planning treatment would be novel for osteoporosis care.

## Supplementary Material

R2_Supplementary_Materials_zjaf139

## Data Availability

Data may be available upon request from the authors. The data that support the findings of this study are available from the corresponding author upon reasonable request for academic studies and are not publicly available due to privacy, ethical, and/or proprietary restrictions.
