# Correction to: Improved prediction of hip fracture using multi-faceted biomechanical computed tomography

**DOI:** 10.1093/jbmr/zjaf163

**Published:** 2025-11-05

This is a **correction** to:

Tony M Keaveny, Annette L Adams, Eric S Orwoll, Sundeep Khosla, Michael R McClung, Mary L Bouxsein, Shireen Fatemi, Bryce A Besler, David C Lee, David L Kopperdahl, Improved prediction of hip fracture using multi-faceted biomechanical computed tomography, *Journal of Bone and Mineral Research*, 2025, zjaf139, https://doi.org/10.1093/jbmr/zjaf139

In the originally published version of this manuscript, the old versions of Figure 1 and Figure 2 were published. In addition to this, an updated Figure 2 was published in the place of Figure 3. This paper has been updated with the correct versions of Figure 1, 2 and 3. The publisher apologizes for this error.

