# Supplementary material for: Improved prediction of hip fracture using multi-faceted biomechanical computed tomography
Source: J Bone Miner Res. 2025 Oct 4;41(5):507–20. doi: 10.1093/jbmr/zjaf139 (PMC13135088; doi:10.1093/jbmr/zjaf139)
Supplement: R2_Supplementary_Materials_zjaf139 [file r2_supplementary_materials_zjaf139.pdf]

SUPPLEMENTARY MATERIALS

Patient Selection

The underlying patient population (**Figure S1**) comprised of all KPSC members aged 65 or older who had any type of abdominal or pelvic CT exam between January 1, 2005 and July 1, 2018. Eligible patients were identified using EHR demographic information and ICD-9/ICD-10 diagnostic and procedure codes, including ICD-9/ICD-10 diagnosis codes for inpatient hospitalizations for any hip fractures. Each patient's observation period started on the date of their CT exam and ended at the earliest of a hip fracture, disenrollment from KPSC, death, 10 years follow up, or the end of data collection (December 31, 2020), whichever came first. Patients were excluded if they: 1) were enrolled in KPSC for less than one year before the CT exam; 2) had any type of hip fracture before the CT exam or a high-energy hip fracture after the CT exam; 3) had a metal implant at the hip; 4) had a diagnosis of an excluded bone pathology (malignant neoplasm of the femur, multiple myeloma, osteomalacia, hypophosphatasia, Paget's disease, osteogenesis imperfecta, or osteopetrosis); 5) were missing information on the CT imaging facility; or 6) had been excluded from the original FOCUS due to a non-usable CT scan. Of the 341,364 patients initially identified, the 271,389 patients (54% women) who met the inclusion and exclusion criteria comprised our eligible population.

This eligible population was then used to construct a case-cohort study sample. Cases were all those in the source population who had a first (fragility) hip fracture during their observation period. A sub-cohort was then selected via random sampling of the eligible population (including all cases), providing an approximate 1:1 ratio of cases to non-cases for each sex. After combining the cases with the sub-cohort and discarding ineligible patients (primarily with incomplete, inaccessible, or unusable imaging data), the resulting "initial analysis sample" comprised of 11,461 patients with complete data (7,913 women, 3,548 men), approximately half with hip fracture. This "initial" case-cohort sample was then further processed to provide the "final" case-cohort sample that was split into a development and validation sets (**Figure S2**, see text for details).

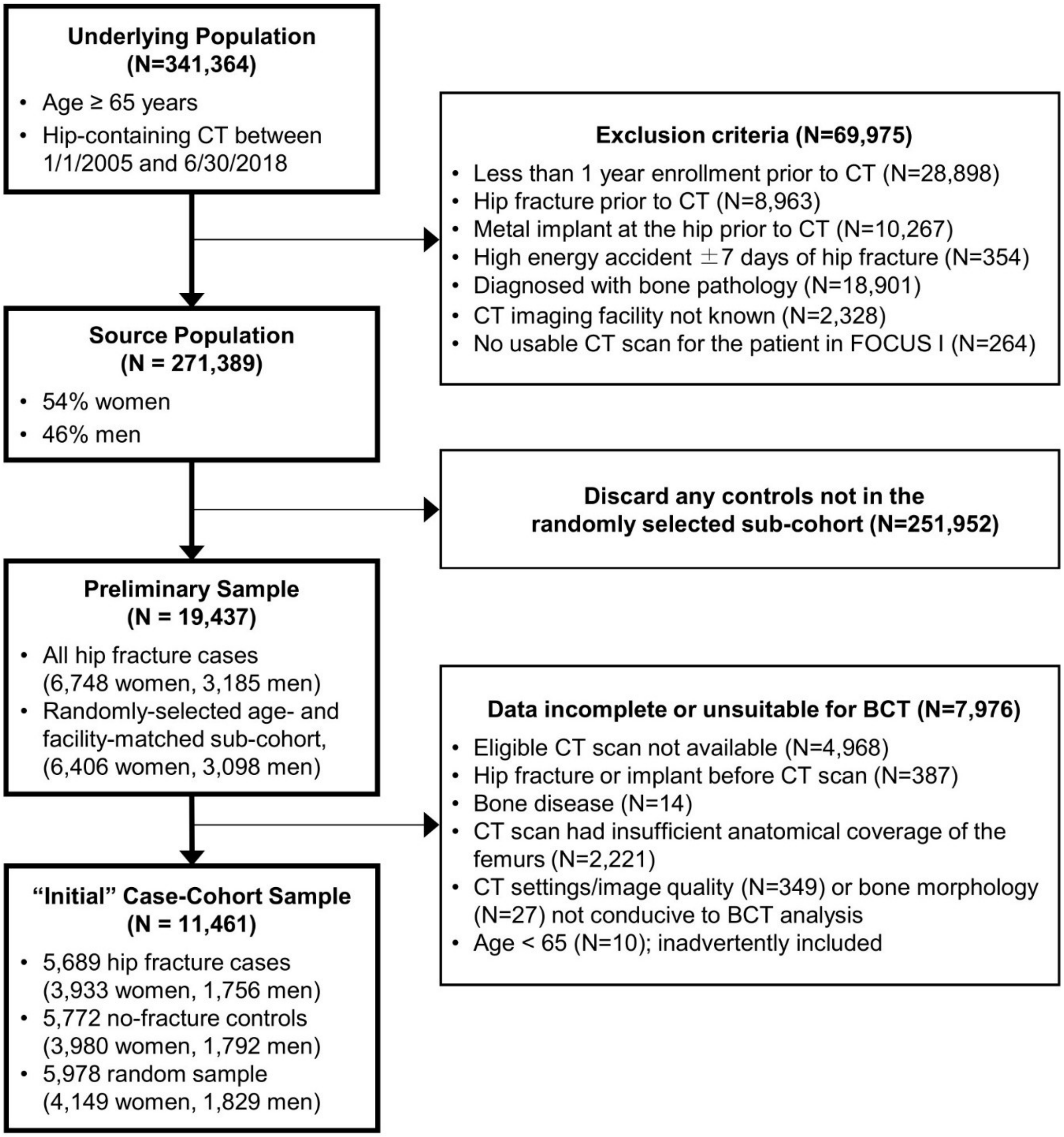

Figure S1: Construction of the “initial” case-cohort analysis sample (N = 11,461 patients) from the underlying population of N = 341,364 patients.

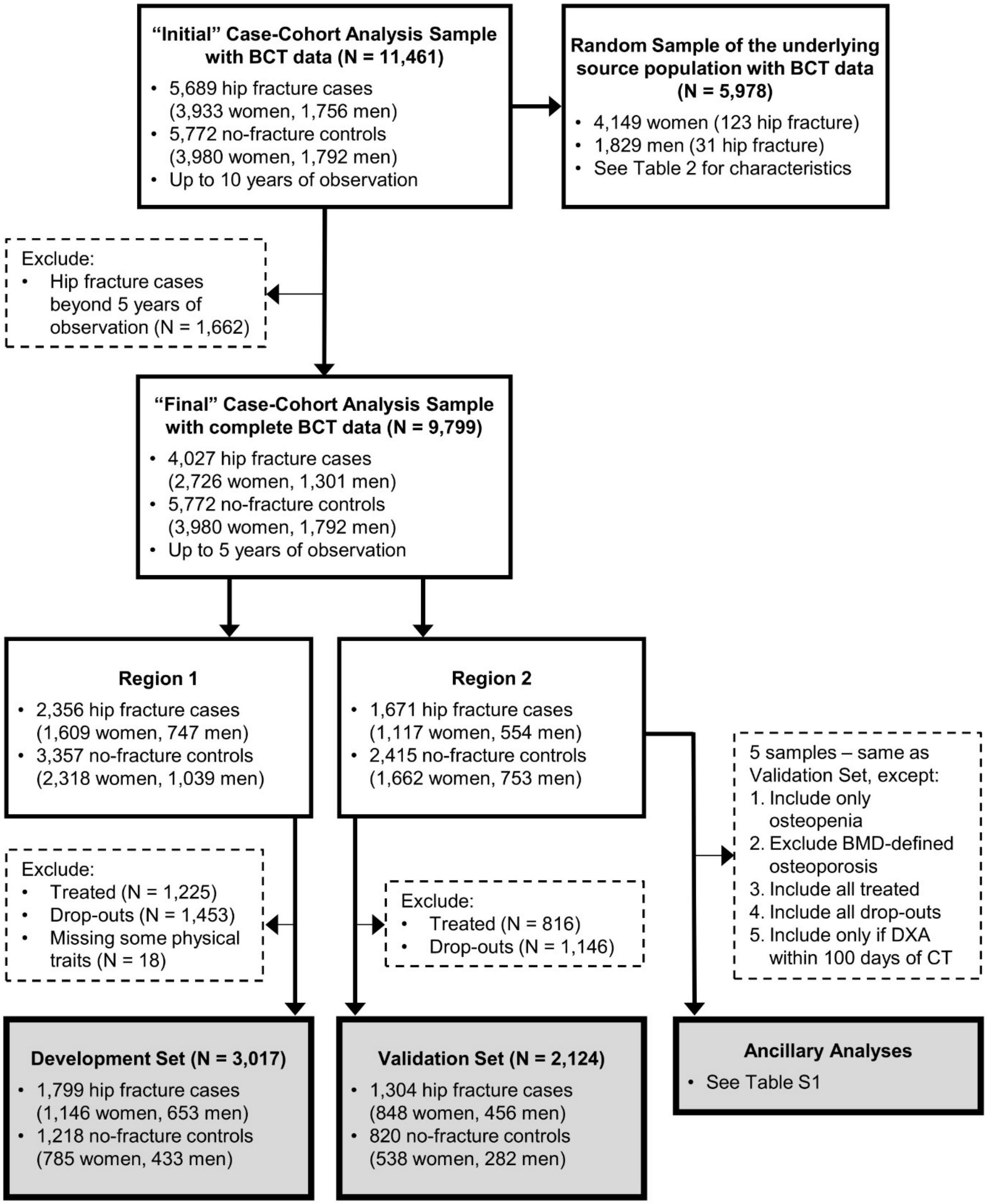

Figure S2: Construction of the development and validation sets used to develop the BCT Risk Score. See Figure S1 for selection of the initial case-cohort analysis sample and the random sample from the underlying population of N = 341,364 patients.

The Hip BMD T-scores for VirtuOst BCT and DXA are Equivalent

Four studies have directly compared hip BMD T-score measurements using VirtuOst BCT from routine clinical CT scans against those using DXA.[1-4] Consistently, the two measurements were shown to be equivalent, falling along the line of Y=X unity and having R^2 values in the range 0.84–0.93. Further, the VirtuOst BMD T-score has consistently predicted incident hip fracture in two large observational studies[5,6] and did so equivalently to DXA.[6] A clinical trial emulation study demonstrated that the VirtuOst BMD T-score identified high-risk patients who benefited from osteoporosis drug treatment for preventing a hip fracture and did so equivalently to DXA.[7,8] Finally, the VirtuOst hip BMD measurements are highly robust to CT scanning variations, for example showing a strong correlation ($R^2 \geq 0.94$) between regular multi-detector CT and low-resolution PET-CT, again in a clinical setting.[9]

For reference, the correspondence (degree of scatter) between BCT vs. DXA is about the same as between left vs. right femur or between Lunar vs. Hologic DXA on the same femur. For example, for 136 women female undergoing colorectal cancer screening by CT colonography and also DXA, during the routine care, the R^2 between their left and right femur by DXA (R^2=0.82) was the same as between their left femur on DXA vs. BCT (R^2=0.84).[2] In a study comparing same-day paired scanning of 90 women on both Lunar and Hologic DXA machines in a research setting, the R^2 was 0.84 for the left femur (all by DXA);[10] this is lower than the R^2=0.92 for the left femur that was observed between same-day scanning of 396 patients for CT vs. DXA in a clinical setting and subsequent BCT analysis, blinded to the DXA data.[4]

These results establish that BCT vs. DXA is at least as consistent as DXA Hologic vs. DXA GE Lunar. In clinical practice, no one distinguishes between left vs. right femur or between Lunar vs. Hologic for diagnosing osteoporosis. In other words, for the purposes of osteoporosis testing in clinical practice, any variations in the hip BMD T-score between left vs. right hip, or between Lunar vs. Hologic, are considered to be clinically acceptable. Given that, for diagnostic purposes, it shouldn't matter clinically if the hip BMD T-score is measured by BCT or DXA.

Intramuscular Fat Measurements

Our method involved two steps for measuring intramuscular fat (adipose) content, which was applied to each individual voxel in the segmented muscle:

1. Adjust the measured Hounsfield Unit value from the CT scan to reflect an assumed (not patient-specific) standardized effective energy (50 keV) and to partially account for the presence of intravenous (IV) contrast during scan acquisition.
2. Using the adjusted HU value, apply a two-component mixture model of pure fat and pure muscle to derive the percent fat content for each voxel. In this mixture model, attenuation is

shared between pure muscle and pure fat based on the attenuation sharing that is expected for the assumed standardized effective energy.

In the first step, the Hounsfield Unit values (as observed on the CT scan) are first adjusted to an assumed effective energy, as follows:

$$HU = CT \times m_{kev} + b_{kev} \qquad \text{[Equation 1]}$$

In this and all following equations, the "*CT*" variable is used for the unadjusted Hounsfield Unit values as measured from the patient's scan, and the "*HU*" variable is used for the adjusted values that are subsequently used in the mixture model. For each individual patient, by applying the above equation to air and fat reference tissues (not in the muscle) in the patient's scan, the patient-specific constants m_{kev} and b_{kev} are calculated as follows:

$$m_{kev} = (HU_{fat} - HU_{air})/(CT_{fat} - CT_{air}) \qquad \text{[Equation 2]}$$

$$b_{kev} = HU_{fat} - (CT_{fat} \times m) \qquad \text{[Equation 3]}$$

in which CT_{fat} and CT_{air} are both measured from the patient's scan (the same values of CT_{fat} and CT_{air} are used for the BMD calibration). Based on the definition of Hounsfield Units, we standardize to HU_{water} = 0 and HU_{air} = –1000. Thus, $HU_{fat} = 1000 \times \frac{\mu_{fat} - \mu_{water}}{\mu_{water} - \mu_{air}}$, where for fat, water and air μ is the linear attenuation coefficient (cm^{-1}), ρ is the density (g/cm^3), $\left(\frac{\mu}{\rho}\right)$ is the energy-dependent mass attenuation (cm^2/g), and $\mu = \left(\frac{\mu}{\rho}\right) \times \rho$. Values for the density and mass attenuation (as a function of keV) can be found in the NIST tables:

https://www.nist.gov/pml/x-ray-mass-attenuation-coefficients,
https://physics.nist.gov/cgi-bin/Star/compos.pl

An additional adjustment is made to help correct for the presence of IV contrast during scan acquisition, which tends to increase the Hounsfield Unit values. The constants $m_{contrast}$ and $b_{contrast}$ used for this non-patient-specific adjustment were obtained from analysis of 60 paired scans taken with versus without contrast:

$$HU = HU \times m_{contrast} + b_{contrast} \qquad \text{[Equation 4]}$$

Here, the *HU* term on the right side is the output of Equation 1, and Equation 4 is only applied if contrast is present. The *HU* term on the left side is the fully adjusted value that is used in the mixture model.

In the second step, the fully adjusted *HU* value of each voxel of muscle is related to a two-component mixture of pure muscle tissue and pure adipose within that voxel in order to calculate a volume fraction of adipose in the voxel ($vf_{adipose}$, ranging from 0 to 1). The following equations describe how the fully adjusted *HU* value of the muscle "mixture" within a single voxel can be broken down into the underlying linear attenuation of the mixture components, namely pure muscle and adipose:

$$HU_{mixture} = 1000 \times \frac{\mu_{mixture} - \mu_{water}}{\mu_{water} - \mu_{air}} \qquad \text{[Equation 5]}$$

As before, $\mu_{mixture} = \left(\frac{\mu}{\rho}\right)_{mixture} \times \rho_{mixture}$ and the attenuation mixture model is:

$$\left(\frac{\mu}{\rho}\right)_{mixture} = \left(\frac{\mu}{\rho}\right)_{muscle} \times mf_{muscle} + \left(\frac{\mu}{\rho}\right)_{adipose} \times mf_{adipose} \qquad \text{[Equation 6]}$$

in which *mf* is the mass fraction. The mass fractions of pure muscle and adipose are calculated in terms of their volume fractions, only one of which is independent in a two-component mixture model. Thus, knowing the left side of Equation 5, the mixture term on the right side of Equation 5 is calculated from Equation 6 (at the assumed value of keV) using the following equations relating density, mass, and volume:

$$\rho_{mixture} = \rho_{muscle} \times \; vf_{muscle} \; + \; \rho_{adipose} \times (1 - vf_{muscle}),$$

$$mf_{muscle} = \frac{\rho_{muscle} \times vf_{muscle}}{\rho_{mixture}}, \; mf_{adipose} = \frac{\rho_{adipose} \times vf_{adipose}}{\rho_{mixture}}$$

The resulting equations are then solved for $vf_{adipose}$ for each individual voxel of muscle. The reported measurement of intramuscular fat content for a patient's muscle is the average value of all voxel values of $vf_{adipose}$ within the segmented muscle.

CT Scanning Protocols

A qualifying CT exam was any type of abdominal-pelvic CT scan identified by CPT code (72192–94, 74150/60/70, 74174–78, 74261–74263, 75635). The CT scans in the initial case-cohort analysis sample (N = 11,461 patients) were acquired in 28 different imaging facilities using 148 different CT scanners, with 28 different types of scanner models (**Table S1**). Almost all scnners were manufactured by GE Healthcare; almost all scans were acquired at 120 kVp and 64% had IV contrast; most scans were reconstructed using a large field of view, a standard reconstruction kernel; slice thickness was in the range 0.5–5.0 mm (70% in the range 2–4 mm). By chance, the development set only contained GE scanners. Otherwise, scanning characteristics were similar between the development and validation sets.

Effects of Scanning Parameters

We performed two different analyses to investigate effects of the main CT-imaging parameters on the overall prediction and the comparison of the prediction between the BCT Risk Score and the BMD T-score. We assessed slice thickness, presence of IV contrast, and reconstruction orientation. For slice thickness, we defined "thick" as above > 3.0 mm and "thin" as ≤ 3.0 mm; for reconstruction orientation there were three levels: the standard transverse orientation, and either coronal or sagittal orientations. In clinical care, scans are reconstructed for archiving using thick sections and/or alternative orientations to reduce file size; some scans are reconstructed in non-transverse orientations to assist in anatomic visualization.

Table S1: Details on CT scanner equipment and acquisition and reconstruction characteristics (percentage of patients unless noted otherwise) for the development (N=6,692 patients) and validation (N=4,769 patients) sets.

Scanning Characteristic	*Development Set*	*Validation Set*
**Sample size**	*6692*	*4769*
**CT Scanner Manfacturer**		
GE Healthcare	100	94.5
Siemens	0	1.3
Canon (Toshiba)	0	2.3
Philips	0	1.9
**CT Scanner Models ***		
GE Healthcare	10	15
Siemens	0	6
Canon (Toshiba)	0	2
Philips	0	4
**CT Scan Type**		
Abdominal	3.7	2.4
Pelvic	18.7	17.3
Abdominal-Pelvic	77.0	78.8
Other abdominal/pelvic	0.6	1.5
**Intravenous Contrast**		
With	60.0	69.6
Without	40.0	30.4
**Tube Current (kVp)**		
100	0.3	1.6
120	97.0	95.2
130	0.0	0.1
140	2.7	3.1
**Kernel**		
Standard or Equivalent	98.9	95.4
Other	1.1	4.6
**Slice Thickness**		
Thin (≤ 3mm)	77.2	88.2
Thick (> 3mm)	22.8	11.8
**Reconstruction Orientation**		
Transverse	56.4	46.1
Coronal	36.2	42.8
Sagittal	7.4	11.1

* number of different scanner models per manufacturer.

In our first analysis, for the development set (N = 3,017 patients) we used a multivariable logistic regression model to assess how including contrast (yes/no), slice thickness (thick/thin), and reconstruction orientation (transverse/coronal/sagittal) into the formulation of the continuous BCT Risk Score could change the AUC value of the overall prediction. In our second analysis, for the main analysis for the validation set (N = 2,124 patients) we ran stratified analyses based on contrast (yes/no), slice thickness (thick/thin), and reconstruction orientation (transverse/coronal/sagittal). Here, we repeated the main comparison of AUC values between the BMD T-score and the BCT Risk Score, but separately for the stratified subcohorts.

For the overall formulation of the BCT Risk Score in the development set, including contrast, slice thickness, and reconstruction orientation together into the formulation only changed the AUC by 0.0007 points, with none of these new terms reaching statistical significance (contrast, $p = 0.10$; slice thickness, $p = 0.71$; reconstruction orientation, $p = 0.16$). Such a small change in AUC means that accounting for these imaging parameters in the formulation would only have a negligible effect on the ROC curve and overall prediction, and thus sensitivity and/or specificity. This lack of effect is likely because the variation in morphology across patients is huge compared to any measurement errors introduced by variations in these scanning parameters.

Consistent with that finding, for the main analysis the comparison of AUC values between the BMD T-score and the BCT Risk Score remained highly significant ($p < 0.0001$) regardless of stratifying by these imaging parameters (**Table S2**). It was not possible to determine if any difference in AUC values between the stratified subcohorts was a cohort or imaging effect.

Table S2: Effects of various imaging parameters on the overall prediction. Pooled sexes (N = 2,124 patients, as in the main analysis).

	Sample Size	AUC [95% confidence interval]		p-value
		BMD T-score	BCT Risk Score	
**Intravenous Contrast**				
With	1456	0.792 [0.768–0.814]	0.874 [0.855–0.891]	< *0.0001*
Without	668	0.827 [0.792–0.857]	0.883 [0.854–0.907]	< *0.0001*
**Slice Thickness**				
Thin (≤ 3mm)	1868	0.800 [0.780–0.819]	0.873 [0.857–0.889]	< *0.0001*
Thick (> 3mm)	256	0.832 [0.773–0.878]	0.909 [0.861–0.941]	< *0.0001*
**Reconstruction Orientation**				
Transverse	969	0.818 [0.789–0.843]	0.881 [0.858–0.902]	< *0.0001*
Coronal	935	0.799 [0.769–0.825]	0.878 [0.854–0.899]	< *0.0001*
Sagittal	219	0.803 [0.739–0.854]	0.878 [0.825–0.917]	< *0.0001*

* p-values compare the AUC values (BMD T-score vs BCT Risk Score) using DeLong's method.

Repeatability Analysis

The measurement repeatability of the BCT Risk Score was assessed for 40 patients who were randomly selected from the development set. Working from the output of a *VirtuOst* BCT analysis of femoral strength, two technicians at O.N. Diagnostics independently measured the new physical traits for each of the 40 patients, blinded to each other, and values of the BCT Risk Score were calculated for each of the 40 patients for each technician. Inter-operator precision of the BCT Risk Score (as a continuous variable) between the two technicians was calculated as the root mean square standard deviation of the differences (SD_{RMS}) in the 40 paired measurements; the binary classifications for high-risk status (BCT Risk Score ≥ 75) were also compared.

Results indicated that the root mean square standard deviation of the differences of the BCT Risk Score between the two technicians was 0.2 units (on a scale of 0–100), equivalent to a coefficient of variation of 0.4% and less than the reporting precision (nearest integer) for this measurement. Orthogonal regression between the paired measurements indicated no proportional bias (regression slope = 1.00, 95% confidence intervals (CI) = 0.996–1.002) or fixed bias (mean difference = 0.1, p = 0.08). Consistent with that result, there was 100% inter-operator agreement in the binary classification of high-risk patients.

Ancillary Statistical Analyses for Validation

To assess robustness of the validation, six stratified samples in the validation set were also analyzed (no patients from the development set were involved). Given the clinical interest in identifying high-risk patients who do not have osteoporosis, stratified analyses included patients only with low bone mass (aka osteopenia,-2.5 < hip BMD T-score < -1.0), patients without BMD defined osteoporosis (-2.5 < hip BMD T-score), patients aged 70 and older, and patients who had DXA within 100 days of the CT scan. For the latter, comparisons were also made against the existing DXA measurements. Expanding the sample for the validation set from the main analysis, in one analysis the exclusion was removed for patients treated with any osteoporosis medication during the observation period; in another analysis the exclusion was removed for dropout patients, who were then recoded as no-fracture controls.

Results from these ancillary analyses (**Table S3**) support the robustness of the results from the main validation analysis. For the subset of patients with low bone mass, the AUC remained significantly higher (p<0.0001) for the BCT Risk Score; and for the binary classifiers, sensitivity for the BCT Risk Score was still high (73.5% for women, 72.4% for men) with good specificity (73.8% for women, 81.9% for men; by definition, all patients with low bone mass tested negative by the hip BMD T-score). The diagnostic odds ratio for the BCT Risk Score in these patients remained high and statistically significant (women: 7.8, 95% confidence interval: 5.4–11.2; men: 11.9, 6.1–23.2), indicating the BCT Risk Score was effective in stratifying osteopenic patients into different risk categories. Likewise, the AUC was significantly higher for the BCT Risk Score than the hip BMD T-score in the subset of patients without osteoporosis, and also when drug-treated patients or dropouts were not excluded. For the subset of patients (N = 220, sex-pooled) from the main analysis who had a DXA within 100 days of the CT scan, sensitivity, specificity and diagnostic odds ratios were similar between the hip BMD T-score from BCT compared to the BMD T-score from DXA at either the hip or hip/spine; the highest sensitivity for BMD was for DXA hip/spine, which was 30.5 points lower than for the BCT Risk Score (40.5 vs. 71.0, respectively) — consistent with the main analysis — whereas specificity for both measurements was equally high (94.4 vs. 93.1, respectively).

Table S3: Ancillary statistical analyses to demonstrate robustness. Unless noted, all samples were selected from the validation set and all modeling parameters were equal to those in the main analysis. Brackets denote 95% confidence intervals. See text for explanations and Table 3 for any additional legends. For these ancillary analyses, formal statistical testing of AUC values was not performed.

Ancillary Analyses for 5-Year Hip Fracture Outcome								
	AUC		Sensitivity		Specificity		DOR	
Low bone mass (osteopenia)								
**Women (N = 354 FX; 267 No-FX):**								
Hip BMD T-score	0.662	[0.618–0.703]						
FRAX hip	0.747	[0.707–0.784]	65.0	[64.9–65.0]	72.7	[72.6–72.7]	4.9	[3.5–7.0]
BCT Risk Score	0.817	[0.782–0.848]	73.5	[73.4–73.5]	73.8	[73.7–73.8]	7.8	[5.4–11.2]
**Men (N = 221 FX; 72 No-FX):**								
BMD T-score	0.627	[0.551–0.697]						
FRAX hip	0.694	[0.623–0.757]	63.8	[63.7–63.9]	66.7	[66.6–66.8]	3.5	[2.0–6.2]
BCT Risk Score	0.819	[0.760–0.866]	72.4	[72.3–72.5]	81.9	[81.9–82.0]	11.9	[6.1–23.2]
Not osteoporosis								
**Women (N = 437 FX; 510 No-FX):**								
Hip BMD T-score	0.728	[0.695–0.758]						
FRAX hip	0.791	[0.762–0.818]	55.6	[55.6–55.7]	84.7	[84.7–84.7]	6.9	[5.1–9.4]
BCT Risk Score	0.853	[0.828–0.876]	65.2	[65.2–65.3]	85.1	[85.1–85.1]	10.7	[7.8–14.6]
**Men (N = 334 FX; 278 No-FX):**								
BMD T-score	0.757	[0.716–0.793]						
FRAX hip	0.793	[0.756–0.826]	45.5	[45.4–45.6]	90.7	[90.6–90.7]	8.1	[5.1–12.8]
BCT Risk Score	0.838	[0.805–0.867]	55.4	[55.3–55.5]	93.2	[93.2–93.2]	16.9	[10.1–28.3]
Age ≥ 70 years								
**Women (N = 714 FX; 309 No-FX):**								
Hip BMD T-score	0.798	[0.768–0.826]	51.7	[51.6–51.7]	89.6	[89.6–89.7]	9.3	[6.2–13.7]
FRAX hip	0.822	[0.792–0.848]	82.8	[82.7–82.8]	67.0	[66.9–67.0]	9.8	[7.2–13.2]
BCT Risk Score	0.862	[0.835–0.885]	86.4	[86.4–86.4]	69.3	[69.2–69.3]	14.3	[10.4–19.8]
**Men (N = 368 FX; 150 No-FX):**								
Hip BMD T-score	0.810	[0.768–0.846]	28.5	[28.5–28.6]	97.3	[97.3–97.4]	14.6	[5.3–40.4]
FRAX hip	0.818	[0.776–0.854]	65.0	[64.9–65.0]	82.0	[82.0–82.0]	8.4	[5.3–13.5]
BCT Risk Score	0.867	[0.833–0.896]	73.1	[73.1–73.2]	87.3	[87.3–87.4]	18.7	[11.0–31.9]
Include all drug-treated patients								
**Women (N = 1,099 FX; 789 No-FX):**								
Hip BMD T-score	0.772	[0.751–0.793]	50.3	[50.3–50.4]	86.2	[86.2–86.2]	6.3	[5.0–8.0]
FRAX hip	0.807	[0.786–0.826]	77.7	[77.7–77.7]	68.6	[68.5–68.6]	7.6	[6.2–9.4]
BCT Risk Score	0.854	[0.836–0.871]	82.0	[82.0–82.0]	70.5	[70.4–70.5]	10.9	[8.7–13.5]
**Men (N = 553 FX; 332 No-FX):**								
Hip BMD T-score	0.796	[0.765–0.824]	29.5	[29.4–29.5]	96.7	[96.7–96.7]	12.2	[6.5–22.9]
FRAX hip	0.818	[0.788–0.844]	63.3	[63.2–63.3]	83.1	[83.1–83.2]	8.5	[6.1–11.9]
BCT Risk Score	0.865	[0.840–0.887]	69.3	[69.2–69.3]	88.6	[88.5–88.6]	17.4	[11.9–25.6]
Include all dropouts								
**Women (N = 837 FX; 1,299 No-FX):**								
Hip BMD T-score	0.760	[0.738–0.780]	47.8	[47.8–47.8]	86.1	[86.1–86.1]	5.7	[4.6–7.0]
FRAX hip	0.792	[0.772–0.810]	75.9	[75.8–75.9]	68.8	[68.8–68.8]	6.9	[5.7–8.5]
BCT Risk Score	0.824	[0.806–0.841]	81.4	[81.3–81.4]	68.8	[68.8–68.9]	9.6	[7.8–11.9]
**Men (N = 456 FX; 678 No-FX):**								
Hip BMD T-score	0.767	[0.738–0.794]	26.8	[26.7–26.8]	94.8	[94.8–94.9]	6.7	[4.5–10.0]
FRAX hip	0.789	[0.761–0.814]	58.1	[58.1–58.2]	81.3	[81.2–81.3]	6.0	[4.6–7.9]
BCT Risk Score	0.799	[0.772–0.824]	66.9	[66.8–66.9]	80.7	[80.7–80.7]	8.4	[6.4–11.1]
DXA within 100 days of the CT scan								
**Both Sexes (N = 148 FX; 72 No-FX):**								
BMD T-score (DXA hip/spine)	0.814	[0.751–0.865]	40.5	[40.4–40.7]	94.4	[94.4–94.5]	11.6	[4.0–33.5]
BMD T-score (DXA hip)	0.818	[0.755–0.867]	39.2	[39.1–39.3]	95.8	[95.8–95.9]	14.8	[4.5–49.3]
Hip BMD T-score	0.804	[0.749–0.857]	35.8	[35.7–35.9]	97.2	[97.2–97.2]	19.5	[4.6–82.8]
FRAX hip	0.864	[0.809–0.905]	64.9	[64.8–65.0]	90.3	[90.2–90.3]	17.1	[7.3–40.1]
BCT Risk Score	0.887	[0.830–0.926]	71.0	[70.9–71.0]	93.1	[93.0–93.1]	32.7	[12.3–86.8]

FRAX Calculations

The 10-year probability of hip fracture was estimated for every patient using the online Fracture Risk Assessment Tool (FRAX) for the USA (https://frax.shef.ac.uk/FRAX/tool.aspx). The femoral neck BMD T-score for input was measured using VirtuOst. As recommended, the FRAX calculations were performed by ethnicity group (Black, White, Asian, Hispanic; unknown was assumed to be White). All risk factors for FRAX except for parental history of fracture (which was not available) were obtained from the KPSC medical records; the "alcohol 3 or more units/day" question was marked "YES" if the patient had a diagnosis at KPSC of alcohol abuse. For parental history of fracture, a "NO" was used for all patients per the User Instructions for FRAX when information is missing. Doing so will lead lower the FRAX risk score for those patients with a true "YES" for this factor.

To assess how the FRAX prediction might change when missing information on the parental fracture history, we performed a sensitivity study in which we removed all other risks factors one at a time and assessed how the AUC changed for each individual missing (removed) factor (setting its value as "NO" for all patients). This process was repeated five times, once for each individual binary clinical risk factor (smoking, rheumatoid arthritis, secondary osteoporosis, glucocorticoid use, alcohol); no combinations were explored. The change in AUC due to removal of each individual binary clinical risk factor was calculated. This analysis provides an estimate of how the reported AUC value for the FRAX score would have changed had the information for parental fracture history not been missing.

Results (**Table S4**) indicated that the largest effect occurred when rheumatoid arthritis was missing — doing so decreased the AUC by 0.002 for women (0.849 vs 0.847) and 0.006 for men (0.835 vs 0.829). The magnitude of this change is well over ten-fold smaller than the differences observed between the AUC values for the BCT Risk Score versus FRAX and thus would not compromise that comparison. The FRAX website provides look-up tables to assess risk of fracture without running the calculator. For the binary clinical risk factors, those tables are based on the *number* of risk factors, not the *type*, implying that the effect of each individual binary clinical risk factor on the overall prediction is similar. Our parameter study confirmed that guidance and demonstrated further that missing any one of the non-parental history factors minimally changed the resulting AUC value for the FRAX prediction of hip fracture at five years. We conclude from these results that missing information on parental hip fracture should have only a minor effect on the reported comparison between the BCT Risk Score and FRAX.

Table S4: Effect on the AUC value for FRAX risk score in the main analysis when one additional risk factor was missing from the FRAX calculation (one at a time, no combinations). This analysis was performed for the 1,386 women and 738 men in the main analysis; *n* is the number of patients that were positive for each risk factor.

Additional Missing Risk Factors *	Women (N = 1,386)				Men (N = 738)			
	AUC	*Delta*	*n*	*n (%)*	*AUC*	*Delta*	*n*	*n (%)*
None	*0.849*	—	—	—	*0.835*	—	—	—
Rheumatoid arthritis	0.847	0.002	580	41.8%	0.829	0.006	264	35.8%
Current smoker	0.847	0.002	95	6.9%	0.832	0.003	73	9.9%
Alcohol abuse	0.848	0.001	38	2.7%	0.834	0.001	37	5.0%
Glucocorticoid use	0.849	0.000	170	12.3%	0.833	0.002	74	10.0%
Previous fracture	0.851	-0.002	363	26.2%	0.837	-0.002	143	19.4%

* Parental history of fracture was missing for all patients; Delta = decrease in AUC value compared to missing no additional risk factors (first row).

References

1. Weber NK, Fidler JL, Keaveny TM, Clarke BL, Khosla S, Fletcher JG, et al. Validation of a CT-derived method for osteoporosis screening in IBD patients undergoing contrast-enhanced CT enterography. *Am J Gastroenterol*. 2014;109(3):401-8. https://doi.org/10.1038/ajg.2013.478
2. Fidler JL, Murthy NS, Khosla S, Clarke BL, Bruining DH, Kopperdahl DL, et al. Comprehensive assessment of osteoporosis and bone fragility with CT colonography. *Radiology*. 2016;278(1):172-80. https://doi.org/10.1148/radiol.2015141984
3. Lin JK, Hearn CM, Getzen E, Long Q, Lee DC, Keaveny TM, et al. Validation of biomechanical computed tomography for fracture risk classification in metastatic hormone-sensitive prostate cancer. *Eur Urol Oncol*. 2024;7(4):794-803. https://doi.org/10.1016/j.euo.2023.10.016
4. Hong N, Lee DC, Khosla S, Keaveny TM, Rhee Y. Comparison of vertebral and femoral strength between White and Asian adults using finite element analysis of computed tomography scans. *J Bone Miner Res*. 2020;35(12):2345-54. https://doi.org/10.1002/jbmr.4149
5. Kopperdahl DL, Aspelund T, Hoffmann PF, Sigurdsson S, Siggeirsdottir K, Harris TB, et al. Assessment of incident spine and hip fractures in women and men using finite element analysis of CT scans. *J Bone Miner Res*. 2014;29(3):570-80. https://doi.org/10.1002/jbmr.2069
6. Adams AL, Fischer H, Kopperdahl DL, Lee DC, Black DM, Bouxsein ML, et al. Osteoporosis and hip fracture risk from routine computed tomography scans: The Fracture, Osteoporosis, and CT Utilization Study (FOCUS). *J Bone Miner Res*. 2018;33(7):1291-301. https://doi.org/10.1002/jbmr.3423
7. Keaveny TM, Adams AL, Orwoll ES, Khosla S, Siris ES, McClung MR, et al. Osteoporosis treatment prevents hip fracture similarly in both sexes: the FOCUS observational study. *J Bone Miner Res*. 2024;39(10):1424-33. https://doi.org/10.1093/jbmr/zjae090
8. Blank RD. Trial emulation to improve fracture prevention treatment in men: editorial on ASBMR-24030174. *J Bone Miner Res*. 2024;39(10):1375-6. https://doi.org/10.1093/jbmr/zjae129
9. Schwaiger BJ, Kopperdahl DL, Nardo L, Facchetti L, Gersing AS, Neumann J, et al. Vertebral and femoral bone mineral density and bone strength in prostate cancer patients assessed in phantomless PET/CT examinations. *Bone*. 2017;101:62-9. https://doi.org/10.1016/j.bone.2017.04.008
10. Fan B, Lu Y, Genant H, Fuerst T, Shepherd J. Does standardized BMD still remove differences between Hologic and GE-Lunar state-of-the-art DXA systems? *Osteoporos Int*. 2010;21(7):1227-36. https://doi.org/10.1007/s00198-009-1062-3
